# Youth Participation in Psychological Literature: A Semantic Analysis of Scholarly Publications in the PsycInfo Database

**DOI:** 10.5964/ejop.v15i2.1647

**Published:** 2019-06-07

**Authors:** Iana Tzankova, Elvira Cicognani

**Affiliations:** a Department of Psychology, University of Bologna, Bologna, Italy; Webster University Geneva, Geneva, Switzerland; London School of Economics, London, United Kingdom

**Keywords:** youth, political participation, civic engagement, activism, psychology, semantic content analysis

## Abstract

The topic of youth participation in the public sphere has received increasing attention within recent psychological research. The literature remains somewhat fragmented between different conceptualizations varying in their specificity or broadness. The present study aims to map the current state of debate in psychology regarding youth civic and political participation and to identify the prevalent themes that characterize the research in the discipline from 1990 to 2016. A semantic content analysis with the software T-Lab was performed on a corpus of 1,777 publications retrieved from the PsycInfo database. The results highlight the increasing number of academic contributions on the topic, confirming the growing importance of the issue within psychology. The study sheds light on the spheres of participation, in which the discipline has attempted to make a contribution, namely: traditional and online political context, institutional civic education, adolescent development, and rights-based activism. Moreover, the findings reveal the existing opposing priorities of research that focus either on the explanation of specific forms of involvement or on the formation of future citizens. Within the thematic attention to young people’s civic and political development, there seem to be two general approaches that see youth in divergent ways: as citizens whose civic capacities are to be fostered or as targets for top-down training interventions. This systematic thematic review calls attention to the disparate ways in which youth participation is being addressed in psychology and highlights the need for greater theoretical integration in the field of study.

In recent years, there has been an ever-increasing interest in youth participation both in political discourse and in academic research within various disciplines. It is also a growing topic of interest in different spheres of psychology, including political, social, developmental and community psychology. The discipline has sought to contribute to the understanding of underlying psychological processes of participative activity (e.g., motivations, attitudes, emotions, sense of belonging, efficacy beliefs, perceptions of contextual influences, etc.) and of the implications of engagement for the well-being and development of youngsters. For example, socio-psychological contributions have examined extensively the general processes related to forms of participation, such as collective action and protesting (Fattori, Pozzi, Marzana, & Mannarini, 2015; van Stekelenburg & Klandermans, 2013; van Zomeren, Postmes, & Spears, 2008), as well as volunteering (Omoto & Snyder, 2002). Considerable interest in research with developmental and community focus has been devoted more directly to the nature and significance of different forms of participation adopted by young people, their correlates and effects in terms of sociopolitical development. In this sense, adolescence and young adulthood are regarded as crucial formative periods, in which youth are engaged in the maturing of their identity and their relationship with society (Erikson, 1968; Yates & Youniss, 1999). On the one hand, research has been motivated by the understanding that these periods of socialization are central for the development of sociopolitical attitudes and behaviors in adulthood, thus concentrating on factors that lead to later participation and on related developmental trajectories throughout the life course (Eckstein, Noack, & Gniewosz, 2012; Finlay, Flanagan, & Wray-Lake, 2011; Sears & Levy, 2003; Zaff et al., 2011). On the other hand, civic engagement has also been regarded as an experience that strengthens young people’s resources and capacities. As a consequence, a substantial amount of literature has concentrated on identifying the possible beneficial relations of social participation to positive youth development (Lerner, Almerigi, Theokas, & Lerner, 2005; Lerner, Lerner, & Benson, 2011), as well as to community development and youth empowerment (Evans & Prilleltensky, 2007; Watts & Flanagan, 2007). In general terms, therefore, psychological disciplines have highlighted the importance of understanding and promoting civic participation among young people both for the health of democratic societies and for the healthy development of youngsters themselves.

However, there is lack of a comprehensive systematic view on how the discipline addresses young people’s active involvement in the public sphere. The present study, based on a dissertation research by the first author (Tzankova, 2018), seeks to obtain an overall image of the current prevalent ideas associated with the topic of youth civic and political participation in psychology. Concepts such as activism, political participation, civic engagement, social participation, community participation, active citizenship, and many others, have been used at times synonymously and at times distinctly to denote various ways of getting involved in societal or collective issues. In this sense, there is some ambiguity over how much studies of differently defined forms of participation actually overlap and what degree of specificity carries each term. Many of these concepts have been defined and developed extensively in the fields of political sciences or education studies to then be borrowed and adopted in psychological disciplines along with their underlying assumptions about what kind of participation is relevant and about young people’s active role, in particular. The field of study has, thus, been characterized by lack of clarity on what constitutes youth participation in the public sphere and how it should be approached.

While there has been wide interest in explaining specific institutional and non-institutional forms of political participation, such as voting or activism, the literature has often considered a broader conceptualization of youth participation as more adequate, considering the sociopolitical opportunities present during adolescence and young adulthood. The notion of *civic engagement*, popularized by landmark contributions on the importance of social capital (Putnam, 2000), has been particularly used in research to cover a wide variety of formal and informal activities in interaction with society, making it an all-encompassing umbrella term (Adler & Goggin, 2005). It has been an especially fruitful concept in the context of research on youth civic development (Flanagan, 2003; Sherrod, Flanagan, & Youniss, 2002; Sherrod, Torney-Purta, & Flanagan, 2010) and has been defined as “individual and collective actions designed to identify and address issues of public concern” (American Psychological Association, 2018). The broadness of such an umbrella notion, however, has been criticized for excessive conceptual stretching: “like other buzzwords, civic engagement means so many things to so many people that it clarifies almost nothing” (Berger, 2009, p. 335).

The concept of *active citizenship* is also considered to be closely related to that of civic engagement (Adler & Goggin, 2005; Shaw, Brady, McGrath, Brennan, & Dolan, 2014; Zaff, Boyd, Li, Lerner, & Lerner, 2010) and has been defined in similarly broad terms. Within the context of European policy on citizenship education, the concept has been defined as “participation in civil society, community and/or political life, characterized by mutual respect and non-violence and in accordance with human rights and democracy” (Hoskins & Mascherini, 2009, p. 462). In this conception, too, the activities considered are of great variety—from electoral, activist to community-based and unconventional types. However, a specific emphasis is given on the values that are at the base of such involvement, as these are indicative of the underlying intention behind the definition of the concept, which is related to the promotion of a specific kind of active citizenship within European policy on education (Biesta, 2009).

Adding to the complexity in the study of youth participation is the difficulty to disentangle the competing assumptions about young people within academic research on the issue. As efforts are being concentrated on civic and political education, the peculiar citizenship status of youngsters is brought to the fore. Adolescents, in particular, are not granted full citizen rights until coming of age and are considered to be in a period of developing crucial political competences, thus justifying the greater attention to the impact of socializing factors and processes. There have been, however, criticisms on how young people’s role as actors in the public sphere can often be challenged by normative and adult-centric assumptions in policy and scientific discourses on citizenship (Hart, 2009; Osler & Starkey, 2003; Smith, Lister, Middleton, & Cox, 2005). Youth can often be framed as citizens-in-formation based on a deficit-based model that tends to overlook existing experiences and rights (Osler & Starkey, 2003). Young people themselves, especially adolescents, may be influenced by general attitudes that portray them in negative terms or as lacking the abilities to contribute significantly to society (Camino & Zeldin, 2002; Smith et al., 2005). In this sense, a lot of research has assumed young people to be lacking interest and knowledge and has concentrated on how to promote their development as “good” responsible citizens. As argued by Staeheli, Attoh, and Mitchell (2013), such an approach runs the risk of attempting to “mould” youth into normative and unchallenging active citizens, rather than seeking to foster their autonomy and critical skills in relation to the political sphere. Consequently, different understandings of youth agency evidence the contested nature of the concepts in academic literature related to young people’s active citizenship.

## Aim of the Study

In view of the outlined considerations, the aim of the present exploratory study is to map the study of youth participation in the civic and political sphere within scholarly psychological literature from the last 25 years, in order to gain better understanding of how young citizens and their actions in the public sphere are viewed and theorized in contemporary psychology. We, thus, explore the use of several most prominent key concepts denoting youth participation (“activism,” “civic or political participation,” “civic or political engagement” and “active citizenship”) through textometrical analysis of publication abstracts. In particular, we seek to systematize the current state of psychological debate on the topic of youth participation by: identifying sub-groups of specific thematic patterns that can be identified in the academic production related to the chosen terms; and analyzing the latent organization of the content in terms of relationships between these themes.

## Method

### Procedure

#### Searching Process

A systematic search was carried out in the electronic database PsycINFO, which is one of the most important bibliographic sources for international literature in psychology. Containing more than 4 million records with extensive coverage from the 1800s to the present, the database is one of the most comprehensive in psychological science and related social and behavioral sciences. With this consideration in mind, we assumed that the resulting references would be sufficient for a comprehensive view of current literature in the discipline.

The research was carried out on 30 August 2016, using each of the following terms: “active citizenship,” “civic engagement” or “political engagement,” “civic participation” or “political participation,” “activism.”^i^ The key terms that were used were chosen by the authors as most relevant in youth participation literature that treats particularly the public civic and political sphere. These were required to appear together with words denoting young people: “youth*” or “young*,” “teen*” or “adolescen*” and delimited to titles, abstracts, keywords or subjects.

#### Data Extraction

The references were organized based on the search terms used. Duplicates were removed and the database was screened to remove erratum pieces to articles and book reviews, deemed not to be original contributions to the scholarly discourse. Moreover, 103 references published before 1990 were excluded from the analysis, resulting in a final database of 1,777 publications published between 1990 and mid-2016. This selection was related to the goal of the present contribution, which was to provide an image of recent and contemporary literature on the topic. Moreover, less recent academic works may not be as accurately and comprehensibly indexed in online databases. The textual corpus was created using the abstracts of the contributions, considering that they represent the first communication to the academic public and could thus provide a concise description of the authors’ main ideas. Each record already had an abstract retrieved through PsycInfo. It is important to stress, however, that abstracts are quite limited in length and, so, they are more structured and less thorough than the publication itself. The entries in the final corpus were tagged so as to indicate the year of publication, the type of publication (journal article, book or chapter), the geographical area relative to the institutional affiliation of the first author, and the key terms used to retrieve the publication from the database. The resulting dataset is available as open access data (Tzankova & Cicognani, 2018).

### Analysis

The abstracts of the references resulting from the bibliographic search were analyzed by means of a lexicographic content analysis, which aims to examine the internal structure of a text corpus through the study of its word distribution and word associations. The software T-Lab 9.1 (Lancia, 2014), which is an all-in-one set of linguistic and statistical tools, was used to perform the analysis. The software allows for a variety of text analysis based on word occurrences and co-occurrences (Lancia, 2004) within units of analysis (*elementary contexts*) defined by the researcher. In our case, the analysis was performed on each text record, that is, abstract of the publications. Through T-Lab it is also possible to identify thematic differences in the documents and relate them to external variables by which the text corpus is classified. The variables according to which the entries in the corpus were classified were: key terms used in the bibliographic search; time period of publication; type of publication. In more detail, the corpus of abstracts was analyzed to obtain the main thematic clusters characterizing the corpus and the latent dimensions through which it can be organized. The *thematic document classification* tool was used, which combines cluster and correspondence analysis of each record in the text corpus. In a first phase of the analysis, automatic lemmatization was performed to reduce the corpus words to their respective headwords according to the linguistic vocabulary consulted. Afterwards, the *thematic document classification* module was used to perform unsupervised clustering with the method of bisecting K-means, which consists of the following steps: 1) a data table of corpus documents x lexical units with presence/absence values is constructed; 2) data is pre-processed through a TF-IDF (term frequency—inverse document frequency) normalization and scaling of row vectors to unit length (Euclidean norm); 3) documents are clustered using the measure of cosine coefficients and the method of bisecting K-means; 4) for each of the obtained partitions, a contingency table of lexical units by clusters is constructed; 5) a chi square test is applied to all the intersections of the contingency table; 6) finally, a correspondence analysis of the contingency table of lexical units by clusters is performed (Lancia, 2004).

The thematic clusters identified by this procedure represent *semantic universes* (Reinert, 1983), that identify the specific vocabulary of a group of publications with respect to the others. The correspondence analysis examines the relationships between the resulting vocabularies in latent dimensions that represent the organization of meanings within the overall discourse.

## Results

### Characteristics of the Bibliographic Corpus

**Figure 1:**
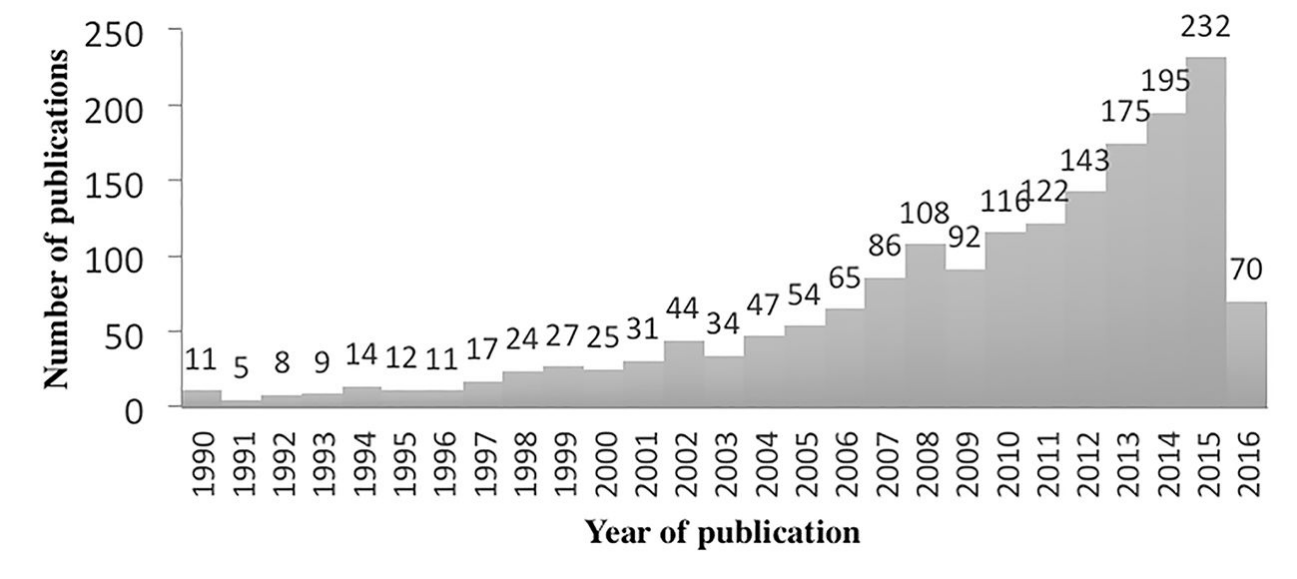
Distribution of publications by year.

Figure 1 shows the temporal distribution of the retrieved publications on youth participation between 1990 and 2016. The increase of literature produced on the topic in the last 10 years is evident. It is worth noticing that the contributions published from 2011 until the date of the bibliographic search account for 52.8% of the whole corpus.

The publications were categorized in six different time periods in order to use the variable in the thematic analysis (see Table 1). We considered the large number of publications in the last 7 years and, while previous years are categorized in periods of 5 years each, the period from 2010 to 2016 is divided in two periods of four and 3 years. Table 2 shows the amount of publications according to their type.

**Table 1:** Number and Percentages of Entries According to Time Period

Time Period	Frequency	Percent
1990–1994	47	2.6
1995–1999	91	5.1
2000–2004	181	10.2
2005–2009	405	22.8
2010–2013	556	31.3
2014–2016	497	28.0
Total	1,777	100.0

**Table 2:** Number and Percentages of Entries According to Type of Publication

Type	Frequency	Percent
Book	51	2.9
Book Section	266	15.0
Journal Article	1,460	82.2
Total	1,777	100.0

**Figure 2:**
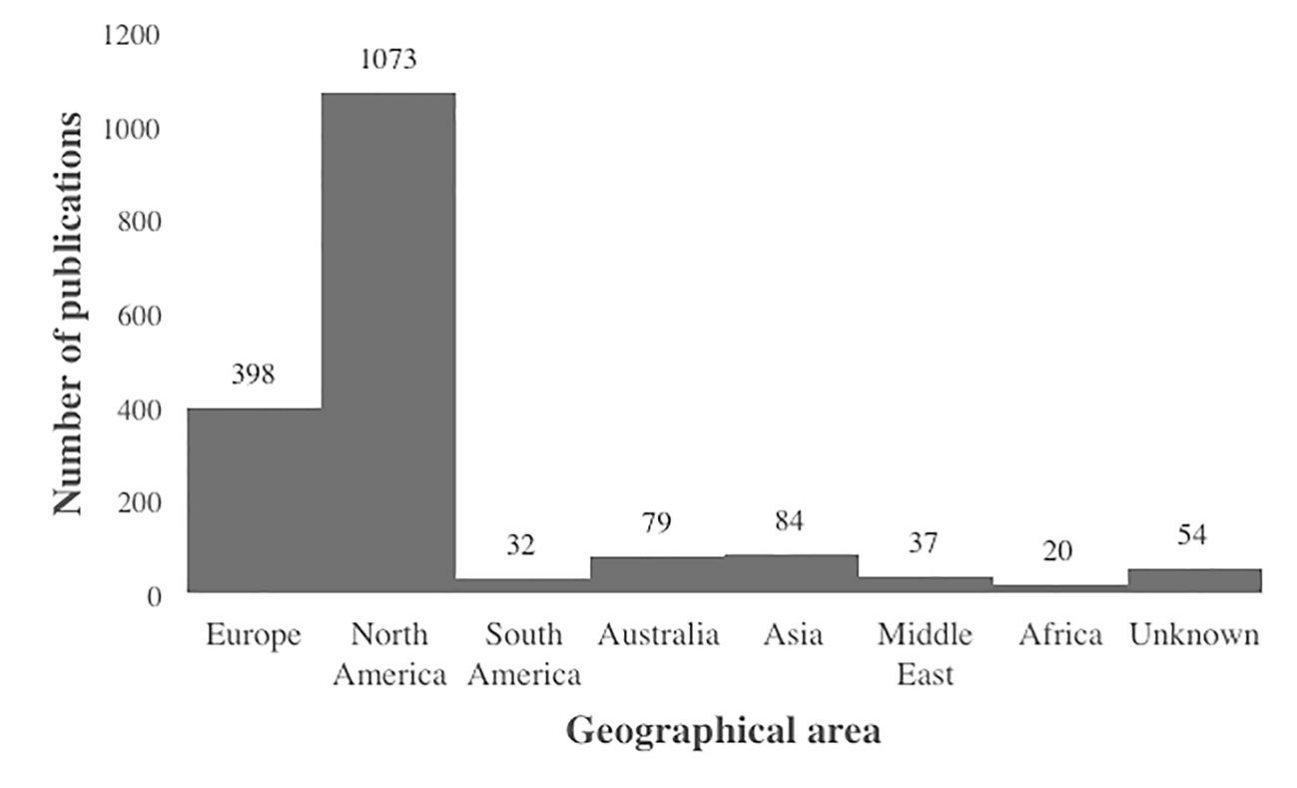
Distribution of publications by geographical area.

Figure 2 shows the distribution of publications according to the geographical area of the first author’s institutional affiliation. The majority of authors were affiliated with an institution in North America (60.4%). For 54 publications it was not possible to retrieve the geographical area of the affiliation from the PsycInfo record, as indicated by the “Unknown” bar in the figure.

The entries were also classified according to the search terms used to retrieve them. The aim was to explore the amount of scientific production related to different keywords denoting citizen participation and their interconnectedness, as seen in Table 3. Contributions could be classified to one term uniquely or, if resulting from multiple searches, to the combination of relevant terms.

**Table 3:** Number and Percentages of Entries According to Search Terms and Their Combinations

Search Terms	Frequency	Percentage
Unique entries		
Civic or political engagement	376	21.2
Civic or political participation	419	23.6
Activism	661	37.2
Active Citizenship	39	2.2
Co-presences		
Civic or political engagement & Civic or political participation	137	7.7
Civic or political engagement & Activism	43	2.4
Civic or political engagement & Civic or political participation & Activism	29	1.6
Civic or political participation & Activism	62	3.5
Active citizenship & Civic or political engagement	4	0.2
Active citizenship & Civic or political engagement & Civic or political participation	1	0.1
Active citizenship & Civic or political participation	6	0.3
Total	1,777	100.0

As shown in Table 3, the term “activism” (37.2%) resulted in the largest amount of scholarly publications, followed by “civic or political participation” (23.6%) and “civic or political engagement” (21.2%). “Active citizenship,” however, yielded limited results (2.2%), indicating that the term has not received wide attention in psychological literature, despite its possible relevance. Moreover, the terms do not seem to be highly related. “Civic or political participation” and “civic or political engagement” obtain the highest number of shared results (7.7%).

### Thematic Classification

The analysis obtained three clusters corresponding to different themes in the analyzed corpus. Each cluster consists of a set of documents characterized by the same patterns of keywords and can be described through the most characteristic lexical units (lemmas) from which it is composed. Chi-square test verifies the significance of a word recurrence within each cluster. Table 4 shows both the percentage of the textual corpus of which each cluster is composed of and a brief list of the most characteristic words for each one. The researchers assigned the names of each cluster after interpreting the main thematic focus of the characteristic lemmas.

**Table 4:** Most Characteristic Lemmas for Each Thematic Cluster (C1-4)

C1: Development of Civic Engagement (36.5%)		C2: Activism (13.2%)		C3: Civic Education as Prevention/Intervention Strategy (21.4%)		C4: Political Participation (28.9%)	
Lemma	χ^2^	Lemma	χ^2^	Lemma	χ^2^	Lemma	χ^2^
youth	918.15	feminist	747.91	health	389.95	political	775.93
civic engagement	233.05	peace	740.87	students	329.06	political participation	672.71
citizenship	193.70	gay	509.76	community	309.46	on-line	498.11
civic	156.36	feminism	361.76	school	299.09	social capital	303.40
development	112.20	protest	356.66	service-learning	273.97	internet	266.75
immigrant	107.92	movement	322.53	program	168.03	news	213.72
ethnic	93.68	war	286.46	tobacco	159.45	Facebook	203.69
young people	78.64	lesbian	270.58	prevention	150.83	offline	177.31
developmental	58.16	women	263.78	African American	134.88	use	171.96
disability	56.93	feminist activism	245.49	teachers	134.53	participation	169.96
identity	56.08	aggression	224.28	alcohol	104.16	voting	169.84
empowerment	53.02	sexual	222.91	service	95.73	social media	169.76
space	47.53	men	215.88	medical	91.45	election	159.74
adolescence	45.24	LGBT	175.68	intervention	89.57	political efficacy	154.37
opportunities	45.24	rights	156.19	educational	81.20	politics	152.53

*Note.* The table reports only the first 15 lemmas according to χ^2^ value (see discussion of the clusters in the following paragraphs for more details on specific vocabulary).

The clusters thus represent domain-specific repertoires that identify different approaches and underlying assumptions to the study of youth civic and political participation. In the following paragraphs the characteristics of each thematic clusters are explored in detail, including—where significant—their relation to the illustrative variables considered in the analysis.

#### Cluster Analysis

##### Development of Civic Engagement (Cluster 1)

The first thematic cluster is the most present one (36.5%) and shows a direct focus on youth (“youth,” “young people”) and adolescents, in particular. It is characterized by words denoting a developmental perspective (“development,” “developmental,” “positive development,” etc.). Moreover, the theme seems to be one of studying civic engagement and active citizenship, as well as associated processes in young people: “civic engagement,” “active citizenship,” “responsibility,” “civic development,” “competence,” “civic knowledge,” “foster.” Based on the interpretation of these semantic patterns, the cluster was named to reflect the core focus on youth development of civic competences. Indeed, the thematic domain seems to pay interest in citizenship education (“citizenship education,” “learning”), but also on creating opportunities for youth empowerment through participatory approaches (“opportunities,” “participatory,” “empowerment,” “power,” “youth-led,” etc.). In this sense, a concern with marginalized groups that may not have equal availability of resources emerges, especially regarding immigrants (“immigrant,” “ethnic,” “disability,” “marginalized”). Book and book section publications characterize the cluster, as well as the search terms “civic or political engagement” (also in combination with “civic or political participation” and “activism”) and “active citizenship.” Moreover, the cluster is characterized by publications in the period between 2010 and 2013.

##### Activism (Cluster 2)

The second cluster (13.2%) represents youth participation in terms of challenging the status quo and of claiming rights related to identity. It is characterized by reference to activist practices of raising one’s voice and defending social causes and rights collectively: “protest,” “movement,” “activism,” “right,” “collective action,” “equality,” “social movement,” “oppression,” “radical,” etc. Participation is conceived as rights-claiming action that challenges inequalities and affirms collective identities, as evidenced by the many references to gender-related terms (“feminist,” “gay,” “gender,” “feminism,” “sexual,” “LGBT,” etc.) and other characteristic identity-related words (“identity,” “assertive,” “social identity,” “identification,” etc.). The focus is on the struggle against injustices and discrimination (“aggression,” “prejudice,” “oppression,” “torture,” “sexist,” etc.) and on the request for rights related to diverse issues (“peace,” “war,” “animal,” etc.). However, the cluster does not seem to be characterized by direct references to young people, rather focusing on marginalized groups and social issues. While both *Cluster 1* and *2* present characteristic words denoting marginalized and socially excluded groups, *Cluster 1* is characterized by lemmas with a focus on developmental processes and fostering of youth citizenship in different targets (adolescents, immigrants). These references are missing in *Cluster 2*, in which the prevalent focus is indicated by a plurality of words denoting existing practices of protest, resistance and identity affirmation that are not specfic to youth only and can be related to a tradition of critical feminist perspectives. This theme is related to results from the search term “activism” and to less recent publications from the 90’s (time periods: from 1990 to 1994 and from 1995 to 1999).

##### Civic Education as Prevention/Intervention Strategy (Cluster 3)

The third cluster (21.4%) emerging from the analysis is characterized by a discourse on participation that relates it to the educational sphere (“students,” “school,” “service-learning,” “educational,” “teachers,” “classroom,” etc.). Youth participation, in this case, is conceived in relation to strategies to be enacted top-down (“program,” “intervention,” “project,” “policy,” etc.), in order to train and promote civic skills (“training,” “skills,” “promote,” “civic responsibility,” “communication skills”), but also to prevent or cope with different health issues or problematic behaviors (“health,” “prevention,” “tobacco,” “alcohol,” “sex education,” etc.). These can also be addressed in the community and local contexts (“community,” “neighborhood,” “community-based”). The cluster is characterized by the search term “civic or political engagement” and publications from North America. While *Cluster 1* also focused on civic engagement and the fostering of certain competences for participation, this thematic grouping does not refer to any developmental issues and remains prevalently focused on strategies and interventions in educational contexts. The main difference between the two clusters seems to be the way that youngsters are intended—tellingly, here the focus is on “students” (seemingly passive recipients), whereas in *Cluster 1* it is “youth” and “young people.”

##### Political Participation (Cluster 4)

Youth participation in the last cluster (28.9%) is related mainly to the political sphere: “political,” “political participation,” “voting,” “political engagement,” “political behavior,” “political activity” and the electoral process (“election,” “campaign,” “party,” “presidential”). Related psychological processes (“political efficacy,” “efficacy,” “trust,” “values,” “attitudes,” etc.) and personality traits (“trait,” “personality”) are brought forward. Interestingly, there is a characteristic attention for the role of media and its digital forms (“on-line,”, “off-line,” “news,” “Facebook,” “social media,” etc.). The focus is on explaining and predicting voting and political engagement in a quantitative research approach (“survey,” “predict,” “effects,” “hypothesis,” “correlation,” “experiment”). This theme, like *Cluster 2*, is also not characterized by references to youth as a group and focus of the research. Likely, the approach in the contributions from this cluster is mostly focused on understanding the underlining factors of participation and young people are the sampled population, but the emphasis is not on them specifically. The cluster is characterized by journal articles and by results from using the search term “civic or political participation” (including in combination with “civic or political engagement” and “activism”).

**Figure 3:**
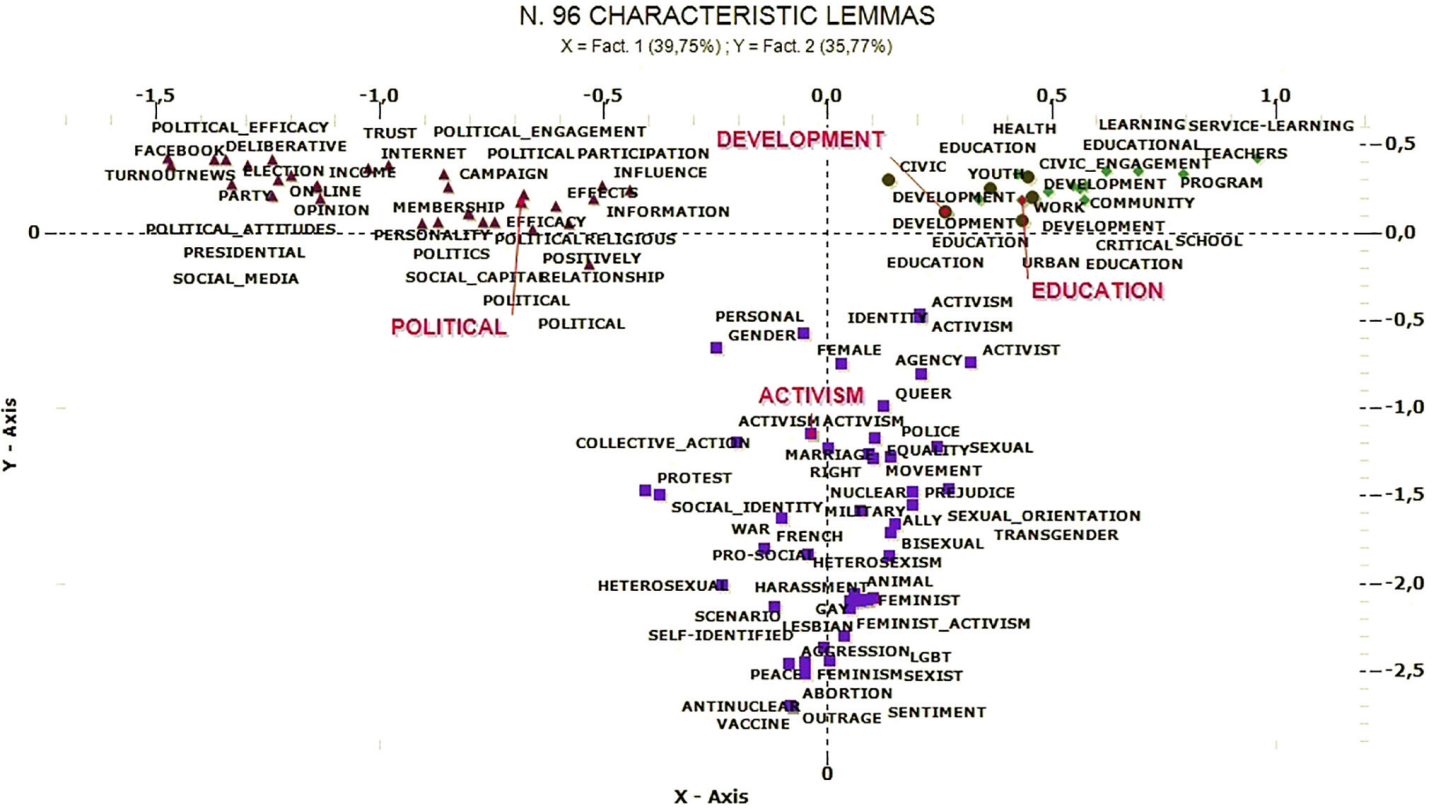
Correspondence analysis: Factor 1 (x-axis) and Factor 2 (y-axis).

**Figure 4:**
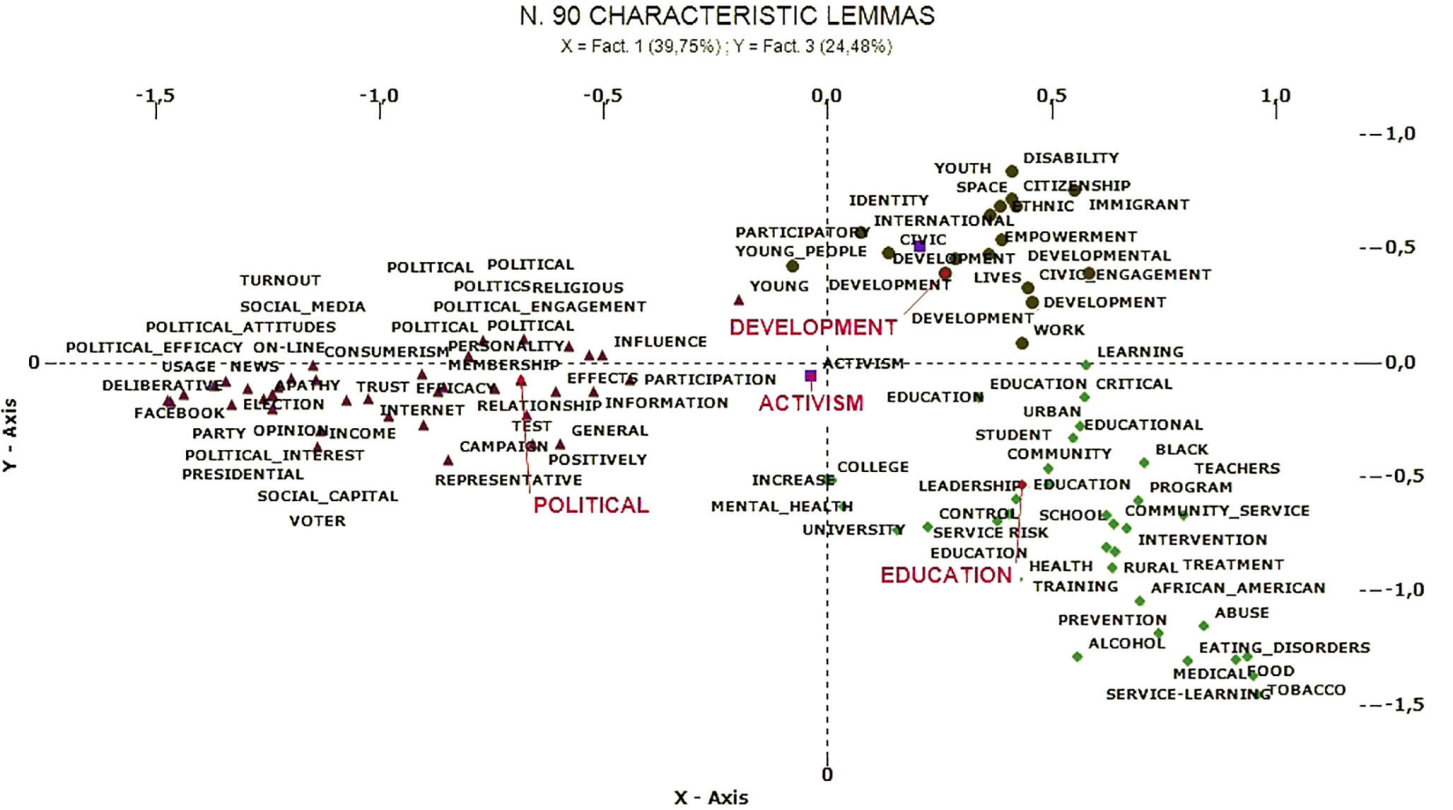
Correspondence analysis: Factor 1 (x-axis) and Factor 3 (y-axis).

Correspondence analysis identified three latent dimensions that organize the corpus, explaining the variance between documents. The thematic clusters are positioned in the factorial space, according to the relative contribution of each as seen in Table 5. Figures 3 and 4 represent the thematic dimensional space and the interactions between the clusters.

**Table 5:** Collocation of the Clusters in the Factors (Absolute Contributions)

Cluster	Factor 1^a^	Factor 2^b^	Factor 3^c^
1: Development of civic engagement	**+0.26**	+0.12	**+0.39**
2: Activism	-0.04	**-1.15**	-0.06
3: Civic education	**+0.43**	+0.19	**-0.53**
4: Political participation	**-0.69**	+0.18	-0.07

*Note.* Values in bold character are above the threshold value of 0.25, equal to 1/*N* (*N* = raws of the analyzed table, cfr. Lancia, 2014).

^a^Youth Participation as Conventional Political Activity vs. Civic Development; ^b^Youth Participation as Rights-Claiming vs. Normative Civic and Political Engagement; ^c^Youth Participation as Empowering Developmental Process vs. Learning and Prevention.

The three factors represent distinctions between different approaches to the study of youth participation in the examined corpus.

#### Correspondence Analysis

##### Youth Participation as Conventional Political Activity Versus Civic Development (Factor 1)

The first factor explains 39.75% of the total variance and differentiates the clusters *Development of civic engagement* and *Civic education as prevention/intervention strategy* from *Political participation*. On the one polarity of the factor, we find references to educational and developmental content (“school,” “civic engagement,” “learning,” “program,” “development,” etc.), while on the other—to conventional political participation (“political participation,” “election,” “on-line,” “social capital,” etc.). The distinction is one between studying the development of civic behavior through educational strategies and analyzing the factors that facilitate political participation.

##### Youth Participation as Rights-claiming Versus Traditional Civic and Political Engagement (Factor 2)

The factor explains 35.77% of the total variance and distinguishes the cluster *Activism* from all the others. Participation of young people intended as active rights-claiming (“feminist,” “activist,” “right,” “identity,” etc.) is opposed to the study of more traditionally promoted civic and political behaviors (“civic engagement,” “political participation,” “school,” “on-line,” “democratic,” etc.). The distinction recalls differentiated attention to non-conformist and conformist action, where the latter is privileged in mainstream research.

##### Youth Participation as Empowering Developmental Process Versus Learning and Prevention (Factor 3)

Explains 24.46% of the total variance and distinguishes the cluster *Development of civic engagement* from *Civic education as prevention/intervention strategy*. On the one hand, the development of youth civic engagement is studied as a positive and empowering process in the transition to adulthood (“youth,” “citizenship,” “civic engagement,” “participatory,” “empowerment,” “adolescence,” etc.). On the other hand, studies are interested in classroom education and prevention (“health,” “school,” “prevention,” “program,” etc.).

## Discussion

The study analyzed scholarly publications in order to map existing conceptualizations of youth participation in the psychological discipline and identify differences and specificities of semantic patterns in the analyzed publications through textometric analysis. The bibliographic research in the database PsycInfo showed how the topic has been the center of an exponential increment in academic interest, especially since 2010. The increase of research on youth participation is in line with the growing attention on fostering citizens’ relationship with the public sphere on the international political stage as evidenced by numerous policies that address the issue in Western democracies. A promising and stimulating development has also been offered by the study of the possibilities that digital participation can bring to the inclusion of young people in the public discourse.

The analysis considered the use of different terms denoting participation to civic and political life and the findings suggest that, within the widespread and growing attention to participation processes in psychological literature, there are marked variations in the ways that youth’s role in contributing to society as citizens is addressed. In particular, the cluster analysis identified four *semantic universes* in the corpus, which suggest the presence of different groupings of publications according to general focus of research: 1) the study of youth as citizens in development, where opportunities and empowerment are to be promoted; 2) the study of youth as actors of social change and rights-claiming in opposition to injustices; 3) the study of educational strategies for promoting civic behavior and preventing risk behavior; 4) the study of predictors and effects of conventional and digital political participation. Correspondence analysis identified three latent dimensions, differentiating the study of youth civic development from that of participation in conventional politics, as well as differentiating the educational focus from the one on empowering developmental processes. Moreover, the research on youth activism in defense of social justice was distinct from the other thematic repertoires.

The analysis was based only on the abstracts and not the full texts of publications, and considered limited number of characteristics, which could not allow us to capture methodological and theoretical aspects of the contributions. Since the abstracts are short and written to resume the main features of a publication, analyzing them could not access the more nuanced aspects of the works. A more in-depth analysis of full texts would certainly evidence more refined linguistic associations and further research could provide a deeper investigation of semantic and thematic patterns associated with different theoretical positions on youth participation, even if based on a smaller amount of publications. However, the analysis of the wide sample of abstracts gathered still offered an extensive overview of understandings of youth participation across a vast sample of academic texts. Moreover, full texts may not always be available for consultation freely as abstracts are. A further aspect to consider is that our corpus was retrieved through the use of only one database, namely PsycInfo, which could over-represent English-language and mainstream psychological research as it indexes a selection of international journals based on quality and relevance. The analysis of the corpus characteristics indeed highlights the prevalence of contributions from North America and Europe, for example. A recommendation for further research using bibliographic records is to consider multiple databases for the data retrieval, but it would also be important for important online databases in psychology to expand collections in order to represent non-English-speaking scholars, as well as open access and non-mainstream research of quality.

Despite these limits, the study evidenced and distinguished the contexts of participation in which psychology has attempted to make a contribution—institutional political sphere, schools and higher education, community contexts and adolescent development, as well as activist movements. The textometrical analysis allowed to shed light on the specific concerns regarding youth participation that characterize each of these areas of study. The findings highlight how current psychological research present two main priorities on youth participation, as two of the publication clusters can be related to a focus on the formation of future citizens (i.e., *Development of civic engagement* and *Civic education as prevention/intervention strategy*) and the other two clusters can be related to a focus on the explanation of existing forms of involvement (i.e., *Political participation* and *Activism*). Each of the identified thematic groups was associated with the specific use of individual terms denoting types of participation. Rather than synonymous, the concepts of activism, political participation and civic engagement seem to reveal particular approaches and focuses of study. Whereas “activism” specified an area of critical research attentive to forms of rights-claiming and justice-oriented collective action, the term “political participation” was most typical in the study of more conventional acts related to the electoral or online political sphere. The latter association points to the conceptual integration of more recent interest in online forms of action within the academic work on traditional political citizenship. Such integration, however, seems less present for justice-based activism, as the publications that were characterized by its study were thematically distinct with respect to the rest of the corpus and were more typical for the 90’s psychology. The findings highlighted also the growing popularity (especially post-2010) of the study of “civic engagement” in psychology. The thematic area delineated by the use of the term does not seem to be distinguished by reference to other specific participative forms, indeed, pointing to the inclusive and broad nature of the concept (Adler & Goggin, 2005). Rather, this group of publications reveals an approach characterized by the examination of developmental processes in adolescence.

The results also indicate that young people seem to assume different roles in the distinct research areas. In the study of what motivates conventional and online political activity or rights-claiming activism, for example, the specificity of addressing youth as a group was not evidenced by the thematic vocabularies. In these groups of publications, youngsters are more probably studied as the population of interest, in which these disparate forms of participation are occurring, and are not distinct from adults in the theoretical considerations. When young people appear as the central topic in the examined contributions, however, they can be characterized differently—namely, as students whose skills and responsibility are to be trained through educational strategies or as young citizens whose civic capacities are to be developed within adequate contexts and opportunities. The results point to a particular attention to adolescence in these areas of study and to assumptions based on the idea of youngsters as citizens-in-formation (Osler & Starkey, 2003; Smith et al., 2005). The distinction between the two thematic focuses, however, also revealed tensions in addressing citizenship development within psychological research between favoring controlled intervention versus guided emancipation.

Overall, our study offered a systematic thematic review of the current academic debates in psychological disciplines regarding the topic of youth participation. The presented paper can be used as a starting point for orienting future research on youth participation by pointing out commonly explored aspects, as well as existing gaps and lack of dialogue between perspectives. The findings evidenced the macro dimensions that orient research in the interpretation of this complex issue within the growing contribution of psychology. These included the quest of understanding developmental processes in young people’s citizenship in parallel to the study of psychological factors related to forms of youth participation in the political sphere. The evidence suggests the need of greater theoretical integration on the topic that would bridge this gap and take into account both the developmental nature of youth participation and the plurality of its actual expressions, without failing to include critical and activist behaviors. Future research should seek to develop an approach that recognizes the political and social impact of the variety of forms of adolescents’ engagement within their formative experiences of citizenship. Moreover, our findings pointed to the growing isolation of the study of identity-claiming and justice-based activism from all other literature. This distinction points to an ever more prevalent normative ideas of youth participation within psychology, which fail to take into account young people’s capacity to challenge societal status and to act for social change outside of the realm of traditional politics (Banaji & Buckingham, 2013). A further effort is needed from scholars on the topic in order to reach a more comprehensive understanding of youth citizenship that includes creative, non-conforming and disruptive actions within the study of the development of offline and online participation.
